# An online interactive identification key to common pest species of Aspidiotini (Hemiptera, Coccomorpha, Diaspididae), version 1.0

**DOI:** 10.3897/zookeys.867.34937

**Published:** 2019-07-30

**Authors:** Scott A. Schneider, Michael A. Fizdale, Benjamin B. Normark

**Affiliations:** 1 Systematic Entomology Laboratory, USDA, Agricultural Research Service, Henry A. Wallace Beltsville Agricultural Research Center, Beltsville, Maryland, USA; 2 Graduate Program in Organismic and Evolutionary Biology, University of Massachusetts, Amherst, Massachusetts, USA; 3 Department of Biology, University of Massachusetts, Amherst, Massachusetts, USA; 4 School of Natural Sciences, Hampshire College, Amherst, Massachusetts, USA

**Keywords:** Agriculture, armored scale insects, ITS, Lucid, plant quarantine

## Abstract

Aspidiotini is a species-rich tribe of armored scale insects that includes several polyphagous and specialist pests that are commonly encountered at ports-of-entry to the United States and many other countries. This article describes a newly available online interactive tool that can be used to identify 155 species of Aspidiotini that are recognized as minor to major pests or that are potentially emergent pests. This article lists the species and features included with a description of the development and structure of the key. The interactive key is free to access at https://idtools.org/id/scales/aspidiotini/about_index.php.

## Introduction

Armored scales (Hemiptera: Diaspididae) are the largest family of scale insects, accounting for approximately one-third of species diversity in the infraorder Coccomorpha. They are among the most invasive insects in the United States (Miller et al. 2005) and are responsible for considerable agricultural damage, estimated to cost roughly $1–2 billion USD in damage and management expenses each year (Miller and Davidson 2005). They are frequently encountered at ports-of-entry to the U.S. and other countries but are difficult to identify because their morphology is highly derived, and specimens require labor-intensive, skilled preparation as slide mounts. Few systematists are trained in their preparation and identification (Hardy 2013).

The tribe Aspidiotini is one of the larger subdivisions within this family, comprising approximately one-quarter of all described armored scales, ca. 720 species of 2,600 in total (~ 28%) (García Morales et al. 2016). The tribe includes many agricultural pests. Miller and Davidson (1990) compiled a list of global armored scale pests, including 199 species, of which 81 belong to Aspidiotini (41%). They subsequently published a list of pests in the United States (Miller and Davidson 2005), which included 43 species from Aspidiotini of 110 in total (39%). Similarly, Beardsley and Gonzalez (1975) listed what they considered the principal armored scale pests of the world, 14 of 43 are from Aspidiotini (35%). Compared to other armored scale tribes, a disproportionate number of species from this group are pestiferous. From Miller and Davidson’s (1990) list, pests belonging to Diaspidini account for 23% (46) of those listed, Lepidosaphidini account for 18% (35), and Parlatoriini for 7% (13). Tribal classifications follow the current, phylogenetically informed framework proposed by Normark et al. (2019).

The classification of aspidiotine genera does not conform to current estimates of phylogenetic relationships and presents a complex revisionary challenge. Recent molecular phylogenetic estimates have revealed paraphyly of several genera (Schneider et al. 2018), including pest-rich genera, where taxonomic changes could hinder identification efforts. Interactive identification keys are useful tools in this regard because they operate independently of hierarchical classification and are thus robust to changes in combination. Online keys offer the advantage of adaptability to reflect nomenclatural changes and reduce time invested in producing and publishing updated dichotomous keys, which are usually organized around genera and are limited in regional scope. This article describes a newly available online interactive key to 155 commonly encountered species from Aspidiotini. Many of the species included have broad geographical distributions and the tool is intended to be applicable toward an international audience. However, the list is based largely upon quarantine interceptions from the United States; thus, the species representation has some inherent biases. The key is designed for the identification of the adult female stage, which has been cleared of body contents and slide-mounted (see Miller and Davidson 2005; Wilkey 1990 for slide mounting protocols).

**Figure 1.**
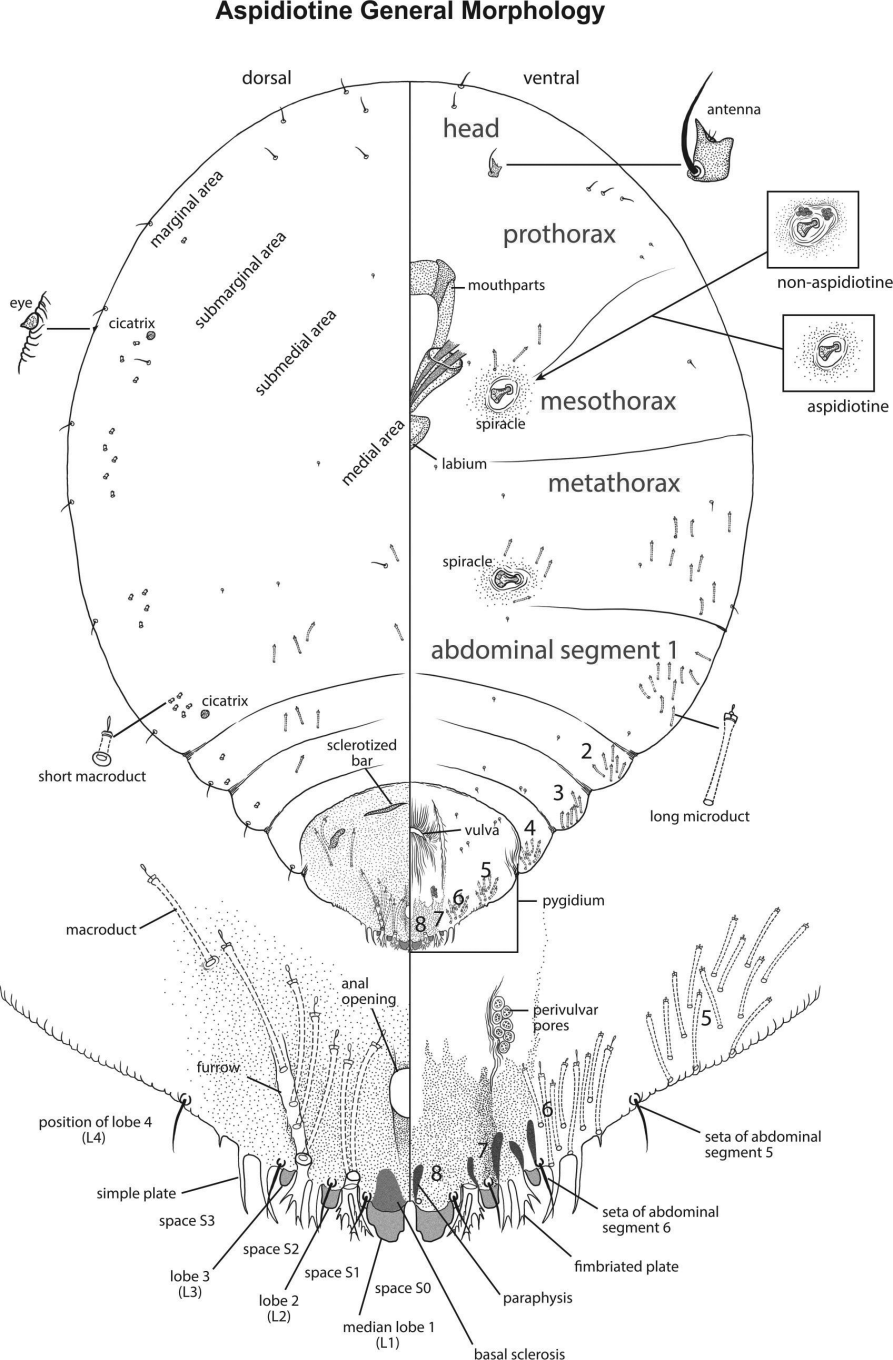
Aspidiotine general morphology. This diagram exemplifies a composite aspidiotine species, illustrating major anatomical features, body segmentation, and traits that a user would encounter in the key. The illustration orients users to the appearance of slide-mounted specimens and terminology used to describe their features. The illustration is based on a similar image presented by Miller and Davidson (2005), their Figure 3. Illustration by Taina Litwak.

The suite of traits that define tribe Aspidiotini includes early paternal genome elimination, one-barred macroduct filaments, one pair of setae on the antennae of adult females, and a lack of pores near the anterior spiracles (see Fig. 1) (Andersen et al. 2010; Schneider et al. 2018). Users should note that the key does not include all species of Aspidiotini and could thus result in false positive identifications if the specimen under consideration is not included. For this reason, it is important to compare the specimen against voucher collections, descriptions, and illustrations. If any question remains regarding the identification of a specimen, a specialist should be consulted. The first version of the key comprises 22% of species in tribe Aspidiotini.

## Project description

The conception of this key is the product of a workshop held at the University of Massachusetts Amherst (UMass) in 2014, organized and hosted by BBN and SAS. Expert identifiers from the United States Department of Agriculture (D.R. Miller), California Department of Food and Agriculture (G.W. Watson, J.W. Dooley), Florida Division of Plant Industry (I.C. Stocks), and Auburn University (N.B. Hardy) were consulted in compiling a list of species and characters for inclusion in the key.

Many of the species (entities) included are commonly considered to be pests (Beardsley Jr and Gonzalez 1975; Miller and Davidson 1990; 2005; Williams and Watson 1988), while several others are recognized as potential emerging pests based on quarantine interception records and input from expert identifiers. The key provides a link for each species to their respective records in ScaleNet (http://scalenet.info/), where the user can find additional information on nomenclatural history, recorded hosts and natural enemies, geographic distribution, references, and additional notes (García Morales et al. 2016). The user is encouraged to regularly check ScaleNet records for up-to-date classification information.

**Figure 2.**
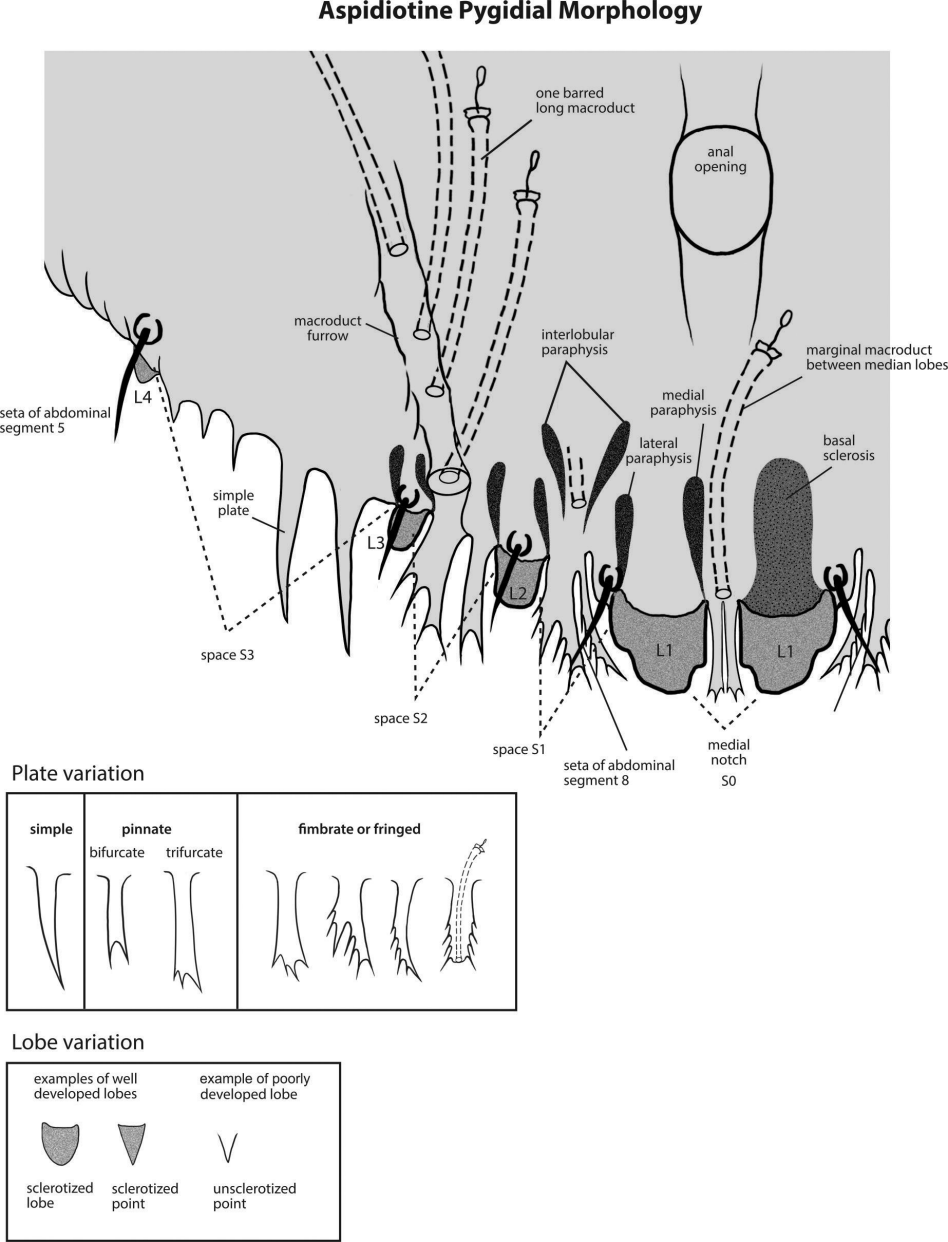
Aspidiotine pygidial morphology. This diagram provides an enlarged view of the general pygidial morphology of aspidiotines. This serves as another guide to the appearance of anatomical features and their terminology. Illustration by Taina Litwak.

The list of characters (features) in this key was composed during the UMass workshop. Some features were adapted from a draft key to the armored scale genera of Australia written by N.B. Hardy. The current version includes 82 features and 195 character states (Table 1). Character states were coded using a combination of published written species descriptions, published illustrations, and slide-mounted voucher material from the entomology collection at UMass and the National Museum of Natural History’s (NMNH) collection in Beltsville, MD. The task of character coding was divided between SAS and MHF; each was responsible for coding a set of characters for all species to establish consistency in the coding scheme. Illustrations and images of character states are included in the key to aid the user in interpreting alternative options. Illustrations of the general morphology of aspidiotines, labeled with descriptive terminology, are provided (Figs 1, 2) in lieu of a glossary.

**Table 1.** Features used to separate species in version 1.0 of the interactive key. Lobes L1–L4 correspond with the pygidial lobes from the median pair through the fourth pair. Interlobular spaces are referred to as S0 (between median lobes), S1 (between median and second lobes), S2 (between second and third lobes), and S3 (between third and fourth lobes).

**Location**	**Features**
General features	Pores near anterior spiracles (presence); body shape (overall); prosoma (constrictions, sclerotized protuberance, processes); lobes L2–L4 (presence); plates / gland-spines (presence); paraphyses (presence, longest length, relative lengths); perivulvar pores (presence); anus (shape, size, relative distance to apex, relative position of vulva); pygidium shape (angle, configuration of apex); dorso-medial pygidial sclerotization (pattern, distinct unsclerotized strip arising from S2, dorsal sclerotized bars near anterior margin of pygidium)
Abdominal segment 8	Lobe L1 (fusion, distance between lobes, orientation, shape at apex, # median notches, # lateral notches); basal scleroses (presence, length, shape); plates S0 (presence); paraphyses S0 (presence, shape); macroduct between L1 (presence, length); dorsal seta of L1 (relative length)
Abdominal segment 7	Lobe L2 (presence, shape at apex, # median notches, # lateral notches); plates S1 (number, shape, fringing, relative length); paraphyses S1 (presence, shape, relative length, relative width); dorsal ducts S1 (pore furrow presence and width, size of orifices, sclerotization of rim)
Abdominal segment 6	Lobe L3 (presence, shape at apex, # median notches, # lateral notches); plates S2 (number, shape, fringing, relative length); paraphyses S2 (presence, shape, relative length, relative width); dorsal ducts S2 (pore furrow presence and width, size of orifices, sclerotization of rim)
Abdominal segment 5	Lobe L4 (presence); plates / gland-spines S3 (number, shape); macroducts S3 (pore furrow presence, marginal ducts presence, size and orientation of marginal ducts, submarginal ducts presence, submedial ducts presence); paraphyses S3 (presence)

### List of terminal taxa included in the current version

*Acutaspis*: *A.agavis* (Townsend & Cockerell), *A.albopicta* (Cockerell), *A.aliena* (Newstead), *A.morrisonorum* Kosztarab, *A.paulista* Hempel, *A.perseae* (Comstock), *A.reniformis* (Cockerell), *A.scutiformis* (Cockerell), *A.umbonifera* (Newstead).

*Aonidiella*: *A.aurantii* (Maskell), *A.citrina* (Coquillett), *A.comperei* McKenzie, *A.gracilis* (Balachowsky), *A.inornata* McKenzie, *A.orientalis* (Newstead), *A.replicata* (Lindinger), *A.taxus* Leonardi, *A.tsugae* Takagi.

*Aspidaspis*: *A.arctostaphyli* (Cockerell & Robbins), *A.densiflorae* (Bremner), *A.florenciae* (Coleman).

*Aspidiella*: *A.hartii* (Cockerell), *A.sacchari* (Cockerell), *A.zingiberi* (Takahashi).

*Aspidiotus*: *A.atomarius* (Hall), *A.cryptomeriae* Kuwana, *A.destructor* Signoret, *A.elaeidis* Marchal, *A.excisus* Green, *A.fularum* Balachowsky, *A.hedericola* Leonardi, *A.kellyi* Brain, *A.nerii* (Bouche), *A.pothos* Takagi, *A.rigidus* Reyne.

*Chentraspisunilobis* (Maskell).

*Chortinaspis*: *C.subchortina* (Laing), *C.subterranea* (Lindinger).

*Chrysomphalus*: *C.ansei* (Green), *C.aonidum* (Linnaeus), *C.bifasciculatus* Ferris, *C.dictyospermi* (Morgan), *C.diversicolor* (Green), *C.fodiens* (Maskell), *C.nepenthivorus* Smith-Pardo, Evans & Dooley, *C.pinnulifer* (Maskell), *C.propsimus* Banks.

*Clavaspidiotusapicalis* Takagi.

*Clavaspis*: *C.coursetiae* (Marlatt), *C.covilleae* (Ferris), *C.disclusa* Ferris, *C.herculeana* (Cockerell & Hadden), *C.subsimilis* (Cockerell), *C.texana* Ferris, *C.ulmi* (Johnson).

*Comstockaspisperniciosa* (Comstock).

*Davidsonaspisaguacatae* (Evans, Watson & Miller).

*Diaspidiotus*: *D.aesculi* (Johnson), *D.africanus* (Marlatt), *D.alni* (Marchal), *D.ancylus* (Putnam) [bark and leaf forms], *D.armenicus* (Borchsenius), *D.caucasicus* (Borchsenius), *D.coniferarum* (Cockerell), *D.degeneratus* (Leonardi), *D.elaeagni* (Borchsenius), *D.forbesi* (Johnson), *D.fraxini* (McKenzie), *D.gigas* (Ferris), *D.hunteri* (Newell), *D.juglansregiae* (Comstock), *D.leguminosum* (Archangelskaya), *D.lenticularis* (Lindinger), *D.liquidambaris* (Kotinsky), *D.maleti* (Vayssière), *D.marani* (Zahradnik), *D.mccombi* McKenzie, *D.osborni* (Cockerell), *D.ostreaeformis* (Curtis), *D.prunorum* (Laing), *D.pyri* (Lichtenstein), *D.shastae* (Coleman), *D.slavonicus* (Green), *D.sulci* (Balachowsky), *D.transcaspiensis* (Marlatt), *D.turanicus* (Borchsenius), *D.uvae* (Comstock), *D.wuenni* (Lindinger), *D.zonatus* (Frauenfeld).

*Dynaspidiotus*: *D.abieticola* (Koroneos), *D.abietis* (Schrank), *D.apacheca* (Ferris), *D.britannicus* (Newstead), *D.californicus* (Comstock), *D.ephedrarum* (Lindinger), *D.rhodesiensis* (Hall), *D.tsugae* (Marlatt).

*Hemiberlesia*: *H.andradae* Okusu & Normark, *H.candidula* (Cockerell), *H.cyanophylli* (Signoret), *H.diffinis* (Newstead), *H.flabellata* Ferris, *H.ignobilis* Ferris, *H.lataniae* (Signoret), *H.mendax* McKenzie, *H.musae* Takagi & Yamamoto, *H.neodiffinis* Miller & Davidson, *H.ocellata* Takagi & Yamamoto, *H.oxycoccus* (Woglum), *H.palmae* (Cockerell), *H.pitysophila* Takagi, *H.popularum* (Marlatt), *H.rapax* (Comstock).

*Lindingaspis*: *L.ferrisi* McKenzie, *L.floridana* Ferris, *L.musae* (Laing), *L.picea* (Malenotti), *L.rossi* (Maskell), *L.williamsi* Balachowsky.

*Melanaspis*: *M.bromiliae* (Leonardi), *M.delicata* Ferris, *M.enceliae* (Ferris), *M.glomerata* (Green), *M.inopinata* (Leonardi), *M.leivasi* (Costa Lima), *M.nigropunctata* (Cockerell), *M.obscura* (Comstock), *M.smilacis* (Comstock), *M.tenebricosa* (Comstock).

*Morganella*: *M.conspicua* (Brain), *M.longispina* (Morgan).

*Mycetaspis*: *M.apicata* (Newstead), *M.defectopalus* Ferris, *M.personata* (Comstock).

*Neoselenaspidussilvaticus* (Lindinger).

*Oceanaspidiotusspinosus* (Comstock).

*Octaspidiotus*: *O.australiensis* (Kuwana), *O.multipori* (Takahashi), *O.stauntoniae* (Takahashi), *O.subrubescens* (Maskell), *O.tamarindi* (Green).

*Pseudischnaspisbowreyi* (Cockerell).

*Rhizaspidiotus*: *R.canariensis* (Lindinger), *R.dearnessi* (Cockerell), *R.donacis* (Leonardi).

*Saharaspisceardi* (Balachowsky).

*Selenaspidus*: *S.albus* McKenzie, *S.articulatus* (Morgan), *S.ferox* Lindinger, *S.kamerunicus* Lindinger, *S.spinosus* Laing.

*Targionia*: *T.arthrophyti* (Archangelskaya), *T.parayuccarum* Munting, *T.vitis* (Signoret).

*Varicaspisfiorineides* (Newstead).

### Technical specifications

**Web location**: https://idtools.org/id/scales/aspidiotini/about_index.php

**Platform**: a website

**Web Server**: CentOS

**Programming language**: PHP 5 and MySQL

**Application version**: 1.0

**Data base**: MySQL

**Data**: 1.0

**Language**: English

**License for use of the key**: Attribution-Non-commercial

**Use of the primary data**: available upon request.

The key was built in Lucid Builder 3.5 (http://lucidcentral.org, Queensland, Australia). It is hosted online on the Identification Technology Program (ITP) webserver (http://idtools.org/) and deployed using a JavaScript Player (Schneider et al. 2019). ITP is part of the USDA Animal and Plant Health Inspection Service Plant Protection and Quarantine division (APHIS PPQ). The key is linked to additional scale insect identification resources available on this platform at http://idtools.org/id/scales/index.php and described in Miller et al. (2014).

The first feature encountered in the key, “pores near anterior spiracles,” will help confirm if a species is included within tribe Aspidiotini. This tool is only suitable for identifying specimens that lack pores near the anterior spiracles. An alternative key for the identification of armored scale genera, developed by J. Dooley and R. Dones (2008), is available at http://keys.lucidcentral.org/keys/v3/Dones_Lourdes/homepage.htm.

**Figure 3.**
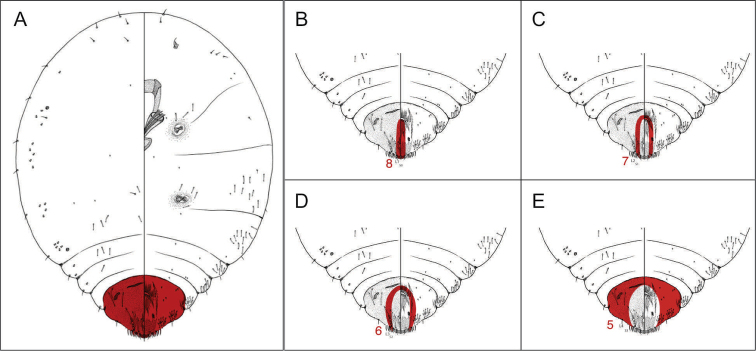
Abdominal segmentation. This diagram shows pygidial segmentation as it is defined for the purposes of this key. The panels highlight (**A**) the pygidium (**B**) abdominal segment 8 (**C**) abdominal segment 7 (**D**) abdominal segment 6 and (**E**) abdominal segment 5. Illustrations by Taina Litwak.

Pygidial segmentation is described and depicted such that each lobe is associated with the interlobular space immediately posterior to it. The pygidial lobes are actually located near the midpoint of each abdominal segment, and each segment encompasses portions of the adjacent anterior and posterior interlobular spaces. This key employs an alternative representation purely for organizational purposes; each abdominal segment is associated with a single lobe and a single interlobular space to avoid confusion. The illustrations of pygidial segmentation (Fig. 3) refer to the region considered for each feature and serve as a guide for the user; note that they are an imperfect depiction of abdominal segmentation as it is usually recognized.

## Conclusions

The interactive key to species of Aspidiotini is useful for identifying major and minor pests as well as several potential pests from this tribe. This key facilitates species-level identification for a challenging group where specialized training and access to uncommon reference materials is usually required. The intent is to provide a user-friendly, accessible tool that simplifies the task of identification by minimizing the need to consult multiple dichotomous keys, which are often limited in zoogeographical or taxonomic scope. The digital format easily allows for updates to the classification, list of features, and species representation in future versions. Species are linked to a continuously updated, comprehensive database of scale insect taxonomic and biological information, ScaleNet. The targeted audience includes an international group of scientists, identifiers at ports-of-entry and government agencies from the local to national level. Both the key and this article are freely available to access.
